# The Current Roadmap of Lung Cancer Biology, Genomics and Racial Disparity

**DOI:** 10.3390/ijms26083818

**Published:** 2025-04-17

**Authors:** Enas S. Alsatari, Kelly R. Smith, Sapthala P. Loku Galappaththi, Elba A. Turbat-Herrera, Santanu Dasgupta

**Affiliations:** 1 Department of Pathology, Frederick P. Whiddon College of Medicine, University of South Alabama, Mobile, AL 36688, USA; esa2222@jagmail.southalabama.edu (E.S.A.); krs808@jagmail.southalabama.edu (K.R.S.); sl1822@jagmail.southalabama.edu (S.P.L.G.); etherrera@health.southalabama.edu (E.A.T.-H.); 2 Mitchell Cancer Institute, University of South Alabama, Mobile, AL 36604, USA; 3 Department of Biochemistry and Molecular Biology, Frederick P. Whiddon College of Medicine, University of South Alabama, Mobile, AL 36688, USA

**Keywords:** lung cancer, racial disparities, NSCLC, SCLC, LUAD, LUSC, genetic mutations, mitochondrial alterations, immune alterations, risk factors, molecular subtypes

## Abstract

Globally, lung cancer is the most prevalent cause of cancer-related death. There are two large histological groups of lung cancer: small-cell lung cancer (SCLC) and non-small-cell lung cancer (NSCLC). Based on histopathological and molecular features, adenocarcinoma (ADC) and squamous cell carcinoma (SCC) are the two major histologic subtypes of NSCLC. Various epidemiological and environmental factors are linked with an increased risk of lung cancer. However, these risk factors show disparities in patients with divergent racial and ethnic backgrounds. Interestingly, different populations were found to harbor distinct molecular features as evidenced by variations in genetic mutation profiles. Moreover, diverse histological and molecular progression patterns are identified in lung cancer, which could be crucial in improving diagnosis, prognosis, and therapeutic planning. In concert with a plethora of nuclear genetic alterations, mitochondrial alteration, epigenetic reprogramming, microbial dysbiosis, and immune alteration signatures have been identified in various lung cancer types. This review article provides a comprehensive overview of screening tests and the treatment strategies for NSCLC and SCLC, including surgery, radiation therapy, chemotherapy, targeted therapies, and immunotherapies. Through the unification of these diverse aspects, this review article aspires to a complete understanding of lung cancer’s genomics, biology, microbial landscapes, and racial disparity and seeks to understand the essential role of racial and ethnic factors in lung cancer occurrence and treatment.

## 1. Introduction

Lung cancer ranks as the second most prevalent malignancy, with an 11.4% incidence rate [1]. Over 230,000 new cases were detected in the United States in 2018, leading to more fatalities than all other cancers including breast, colon, and prostate cancer combined [2]. According to GLOBOCAN 2020 data, approximately 2.3 million new cases (11.4%) and almost 1.8 million deaths from lung cancer were recorded in 2020 [3]. Lung cancer is uncommon before the fifth decade of life, but its incidence rises with age [3]. In the U.S., lung cancer incidence among males continues to decline, while females showed an initial increase followed by a decline. It is particularly marked among younger women who have recently demonstrated higher incidence rates than males, notably for non-Hispanic Whites and Asians/Pacific Islanders [4]. Similarly, a study by Nolen et al. reported higher rates of lung cancer in the United States in young women than men of similar age, extending to those aged 50–54 [5]. A very recent study showed reductions in lung cancer mortality rates that have exceeded reductions in incidence, particularly among men (5.0% vs. 2.6% annually) and women (4.3% vs. 1.1% annually) [6]. On the other hand, the disparity in lung cancer incidence still exists among various racial and ethnic groups. The highest incidence rates and the slowest decline were seen in Native Americans, with various States, including Mississippi and Kentucky, continuing to experience mortality rates two to three times higher than most Western States due to historic smoking prevalence [6]. In addition, Cuban males show higher incidence rates among other Hispanic groups, whereas U.S.-born Black males show higher incidence rates than Caribbean-born Blacks [7]. Among females, US-born Blacks exhibit the highest incidence rates [7]. Nevertheless, lung cancer is the most common cause of cancer-related death in all parts of the world [8]. In 2020, lung cancer was responsible for around 1.8 million deaths, accounting for 18% of all cancer deaths. The age-standardized mortality rate (ASMR) was 18.0 per 100,000 (25.9 in men and 11.2 in women) [9,10]. Mortality exhibits substantial regional variation. The highest rates are seen in countries with high Human Development Index (HDI) scores, primarily from Europe and North America, while the lowest rates are noted among those mainly located in Sub-Saharan Africa [9].

In this review article, we summarize the current knowledge of lung cancer and racial disparities, focusing on various aspects, including genomics, biology, and microbial landscapes. We also presented divergent histopathological and molecular subtypes, epidemiology and risk factors, histopathological and molecular progression patterns, nuclear and mitochondrial genetic alterations, epigenetic alteration, immune system dysfunction, and microbiome dysbiosis associated with lung tumorigenesis.

## 2. Epidemiology and Risk Factors: An Ethnic and Ancestral View

### 2.1. Histological Subtypes of Lung Cancer

Lung cancer is divided into two major groups: small-cell lung cancer (SCLC) and non-small-cell lung cancer (NSCLC). SCLC is aggressive and has a high risk for distant metastasis at initial diagnosis [11] and accounts for 12% of all lung cancer cases [12]. NSCLC is, conversely, the most common group, representing 80% to 85% of the lung cancer cases [13]. Among the NSCLC, adenocarcinoma (LUAD) is the most common histologic subtype, accounting for 45% of all cases, followed by squamous cell carcinoma (LUSC) at 21% of cases, while 23% attributed to unclassified histologic subtypes [12]. Notably, LUAD is more common in never-smokes with a predominant EGFR gene mutation, whereas LUSC is more common among smokers with a predominant TP53 gene mutation [14,15].

### 2.2. Environmental and Lifestyle Risk Factors

Epidemiology and risk factors involve a complex interaction of environmental, genetic mutations, and lifestyle factors that contribute to susceptibility to lung cancer and its outcomes [16,17]. However, these interactions become more significant when considering ethnic and ancestor differences. Although smoking is the predominant cause of lung cancer, another study revealed that 10–25% of all lung cancer patients have never smoked [18]. This disparity underscores the need to investigate additional risk factors other than smoking, especially in populations where lung cancer is not related to smoking [19]. Cigarette smoking remains a major risk factor for lung cancer development. The initiation of smoking habits is mediated by peer pressure, family habits, and psychological distress [20,21]. Interestingly, Harrell et al. reported that demographic factors such as race, socioeconomic status, and pubertal development were significant predictors of early smoking initiation among schoolchildren [22]. Additionally, preventative measures for air pollution include techniques like urea-selective catalytic reduction (SCR), diesel particulate filters, and NOx storage-reduction catalysts approved to enhance air quality to avoid additional health effects from gaseous as well as particulate air pollution pollutants [23]. As a reason, there were substantial declines in lung cancer incidence in the USA from 2007 to 2018. On the other hand, there has been little change in rates among never-smokers, though rates increased significantly in Asian and Pacific Islander populations [24]. In Denmark, lung cancer trends are influenced by historical smoking patterns, where a decline in male smoking rates led to reduced incidence. In contrast, the prevalence of smoking in women remained stable for longer, contributing to a later increase in lung cancer incidence [25]. Existing evidence suggests that passive smoke is the cause of a significant proportion of lung cancer in women. For instance, Du et al. reported that passive smoking accounts for about 17.9% of lung cancer cases among never-smoking women, most of them exposed to household smoking [26]. Moreover, a study of Moroccan women showed that 75% of lung cancer cases were recorded in never-smokers, and LUAD was the most common subtype among passive smokers [27].

Zhu et al. reported that non-smoking people who drink tea ≥ 2 cups/day have a greater risk of lung cancer [28]. At the population level, cigarette smoking is the primary determinant of the occurrence of lung cancer [29]. Environmental factors increase the risk of developing lung cancer, such as air pollution, occupational exposure, secondhand smoke, and radiation exposure [30,31]. In China, a study by Liu et al. observed that occupational environment and meteorological conditions synergistically affect lung cancer development [32]. Furthermore, Chinese-style cooking increases lung cancer risk [33]. Moreover, long-term exposure to air pollutants such as PM2.5, NO_2_, and NOx significantly increases the risk of developing lung cancer [34]. The World Cancer Research Fund (WCRF) reported that drinking water with high concentrations of arsenic increases lung cancer risk, and the evidence was reported as “convincing” [35]. Additional interaction of these air pollutants with poor lifestyle and high genetic risk dramatically raises the likelihood of lung cancer occurrence [35]. Similarly, Huang et al. showed the same results [36]. However, predicting cancer associated with environmental factors like alcohol consumption and smoking can alter based on the variation in polymorphism of xenobiotic metabolizing enzymes (XME) genes [37]. Pettit et al. studied the genetic correlation between various traits and lung cancer risk, indicating a negative genetic correlation between lung cancer risk and some traits, including dietary behaviors, fitness metrics, educational attainment, and other psychosocial characteristics. On the contrary, the body mass index (BMI) showed a positive genetic correlation with the likelihood of lung cancer [38].

The relationship between lung cancer risk and dietary items like fruits, vegetables, micronutrients, phytochemicals, fat, and beverages has been studied. An increased intake of fruits, vegetables, and carotenoid-rich foods is associated with a reduced risk of developing lung cancer [35]. On the contrary, higher intake of retinol, red meat intake, processed meat intake, alcohol drinking, and dietary fat have been associated with an increased risk of lung cancer. However, no link has been reported between the phytochemical “bioflavonoid” and lung cancer risk.

### 2.3. Genetic and Racial Disparities and Lung Cancer Susceptibility

Lung cancer risk is influenced significantly by different racial and ethnic disparities. Individuals with African ancestry (AA) have higher mortality rates and incidence of lung cancer development at an earlier age compared to individuals with European ancestry (EA) due to disparities in preventive screening monitoring and treatment disparities [39,40]. In addition, there is a significant disparity in the metabolic pathways and how the body processes nicotine between AA and EA groups, as AA has lower levels of cotinine glucuronidation [41]. Non-Hispanic AA males show the highest rates of mortality and lung cancer incidence compared to all race-ethnicities [42,43]. Similarly, Primm et al. showed persistent disparities in NSCLC incidence between AA and EA men [44]. Interestingly, despite disparities in diagnosis and treatment, AA and Asian NSCLC patients demonstrate better outcomes for the same-stage cancer compared to EA patients [45]. The cause for disparities is genetic ancestry, as AA populations with LUSC have more genomic instability and aggressive molecular traits, while AA patients with LUAD have a higher frequency of *PTPRT* and *JAK2* gene mutations [46,47]. Additionally, the Asian population demonstrates a higher frequency of *STK11*, *TP53*, and *EGFR* gene mutation [48], but they have longer survival rates and higher chemotherapy responses in comparison to EA patients [49]. Another study linked *TP53*, *KRAS*, and *KEAP1* gene mutations with worse overall survival, whereas *EGFR* gene mutations are associated with a higher chance of survival [50]. Recent studies found that EA patients have higher mortality rates compared to Hispanics and Asians, and they have a higher susceptibility to lung cancer due to higher frequencies in smoking-related loci [51,52]. Many studies have revealed racial disparities in the genetic mutation profile of lung cancer patients. Compared to Japanese patients, EA-LUSC patients present a higher frequency of mutations in *TP53*, *PIK3CA*, *KEAP1*, and *NFE2L2* genes [53]. On the contrary, EA-LUAD patients exhibit a significantly lower occurrence of *EGFR* mutation but an increased frequency of mutation in the *PIK3CA*, *KEAP1*, *KRAS*, *TP53*, *BRAF*, *NF1*, *STK11*, *RBM10*, and *MET* genes. Weiner and Winn reported a higher prevalence of *EGFR* gene mutation in the East Asian population and more predominant *KRAS* and *STK11* gene alterations in EA and AA populations [54]. Generally, the disparities in survival rates between EA and AA populations are noticeable in patients who are young and have localized tumors [55]. The disparities also exist in histological subtype, stage, and tumor grade. Asian or Pacific Islander (API) exhibit a higher frequency of adenocarcinoma (ADC) compared to AA, EA, and American Indian/Alaska Native (AIAN) patients [56].

## 3. Current Detection and Treatment Modalities of Lung Cancer

NSCLC treatment varies depending on the tumor stage and overall health [57]. Surgery is a treatment option for early-stage lung cancer. There are four types of resections: wedge resection (removing the tumor and with normal tissue), lobectomy (removal of one whole lobe with the tumor), pneumonectomy (removal of a lung), and sleeve resection (removal of part of the bronchus). Following surgery, some patients may undergo chemotherapy and radiation therapy. Targeted therapy is used to attack cancer cells based on their specific characteristics, including monoclonal antibodies, tyrosine kinase inhibitors (TKIs), as well as mammalian target rapamycin (mTOR) inhibitors that include mTOR, and KRAS G12C inhibitors. By binding on specific sites, monoclonal antibodies can mark cancer cells for their destruction by the immune system. Furthermore, tyrosine kinase inhibitors target specific signals for cancer growth and spread, including specific EGFR tyrosine kinase inhibitors. Indeed, mTOR inhibitors block a protein called mTOR to keep cancer cells from growing and prevent the growth of new blood vessels. KRAS G12C Inhibitor is a drug that blocks transcription of *KRAS* p.G12C and halts the growth of cancerous cells. Immunotherapy treatments such as immune checkpoint inhibitors (ICIs), however, actually boost and restore natural defenses against cancer in patients. PD-1 and PDL-1 inhibitor therapy is a type of immunotherapy that prevents the binding of PD-1 protein on the surface of T cells with PD-L1 protein found on some types of cancer cells, allowing T cells to kill tumor cells [57]. Actually, the ICIs reported an objective response rate of around 42% in NSCLC patients, even higher among those with PD-L1 expression ≥ 50% [58]. Additionally, perioperative immunotherapy, primarily through PD-1/PD-L1 inhibitors, has made landmark strides in resectable NSCLC to establish a new standard of care for stages II to III due to the reduction in postoperative recurrence with an overall survival improvement [59]. The other approach to immunotherapy is a CTLA-4 inhibitor, which blocks CTLA-4 on T cells binding of B7 provided by antigen-presenting cells (APCs) so that T cells can kill the cancer cell [57]. Notably, both smokers and non-smokers with lung cancer benefit from immunotherapy [60]. Another treatment approach uses a laser beam (laser therapy) or drug and a specific type of laser light (photodynamic therapy); however, the drug is not effective until exposed to laser light in photodynamic therapy. Interestingly, fiberoptic tubes carry laser light to the site of cancer cells to activate the drug. Moreover, cryosurgery can freeze and destroy abnormal tissue, such as carcinoma in situ. Electric current is used to heat the needle or probe in electrocautery treatment to destroy abnormal tissues. A variety of treatment options exist for SCLC [57]. Surgery may not be the answer for SCLC, as it may happen bilaterally. Indeed, there is always the possibility of chemo and radiation following surgery. Systemic chemotherapy is a standard treatment for SCLC. In addition, external radiotherapy is given to SCLC patients, which is used as palliative therapy. Another effective option for SCLC treatment is immunotherapy drugs like mepolizumab and Durvalumb. A laser beam is also used to destroy SCLC cells. Interestingly, Personalized medicine in lung cancer, particularly NSCLC, involves tailoring treatment based on patient genomics. Such approaches improve treatment specificity, targeting genetic mutations that confer resistance and deliver nanoparticles to target NSCLC biomarkers [61].

Three lung cancer screening tests have been tested before the onset of symptoms: low-dose computed tomography (LDCT), chest X-ray, and sputum cytology [57]. A very low dose of radiation is used in LDCT to generate a sequence of very detailed pictures by X-ray machine. A chest X-ray uses an X-ray beam to capture images of organs and bones within the chest. Sputum cytology examines the sputum cells by using a microscope to identify abnormalities. These scanning tests are not sensitive enough to detect lung cancer early, as they may produce false negative or false positive results. Also, the chest is exposed to radiation in LDCT and chest X-ray tests. However, chest X-ray and sputum cytology are low-cost, and a combination of both is probably helpful in detecting early lung cancer in high-risk people [62]. CRISPR technology is also being used for biomarker identification in lung cancer patients to allow for better diagnosis and assess prognosis [63]. However, CRISPR-Cas9 is still in the early stages of development, lacking proven clinical protocols, and carries risks of incorrect editing [64].

## 4. Molecular Progression of Lung Cancer

**Figure 1:**
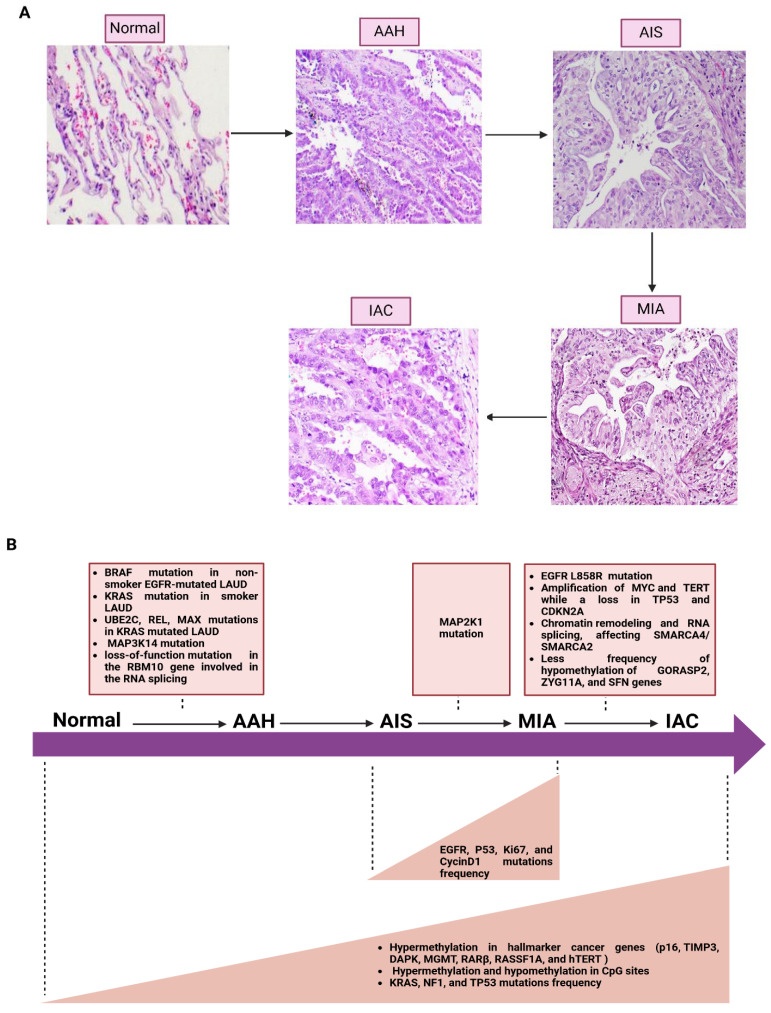
The progression model for lung adenocarcinoma (LUAD). (**A**) The LUAD initiates from atypical adenomatous hyperplasia (AAH), a lesion derived from glandular cells in the epithelial tissue of peripheral airways, to adenocarcinoma in situ (AIS), then minimally invasive adenocarcinoma (MIA) before the next step into invasive adenocarcinoma (IAC). (**B**) The *BRAF* mutation is related to *EGFR*-mutant AAHs, whereas *UBE2C*, *REL*, and *MAX* mutations are related to *KRAS*-mutant AAHs. Additionally, *MAP3K14* mutation and loss-of-function mutation in the *RBM10* gene are disposed of in AAH. The *EGFR* mutation frequency, *p53* and *Ki67*, and *cyclinD1* expression level are higher in MIA than in AIS. Moreover, *MAP2K1* mutation is disposed of in MIA. In addition, the *KRAS*, *NF1*, and *TP53* mutation frequencies, hypermethylation of hallmark cancer genes, hypermethylation, and hypomethylation of CpG sites are significantly elevated from AIS to MIA or IAC compared to AAH. The genetic and epigenetic changes associated with IAC are *EGFR* L858R mutation, amplification of *MYC* and *TERT*, loss of *TP53* and *CDKN2A*, chromatin remodeling and RNA splicing, less frequency of hypomethylation of *GORASP2*, *ZYG11A*, and *SFN* genes. Figure (**B**) was created with https://www.biorender.com/ (accessed on 11 November 2024).

A more profound understanding of the molecular process continuum to lung cancer initiation and progression is urgently needed to improve patients’ prognosis and disease management. Lung cancer initiation is caused by genetic alterations that also drive and maintain lung cancer progression. These can be mutations, epigenetic variations, and alterations in non-coding RNAs or microRNAs. NSCLC is a multistep process ranging from normal epithelium to invasive carcinoma throughout histological stages. Normal epithelium first develops into hyperplasia, characterized by an abnormal proliferation of epithelial cells. Hyperplasia can also progress to metaplasia, during which epithelial cells convert into a different type of cell in response to inflammation or chronic irritation. In such situations, morphological and cell growth abnormalities may progress to dysplasia afterward. Epithelial dysplasia may progress to carcinoma in situ (CIS), a pre-invasive state where atypical cells remain bound by the basement membrane. Ultimately, these changes may progress to invasive carcinoma, where malignant cells cross through the basement membrane [65]. LUAD progresses in a systematic and sequential manner (Figure 1A). It starts from atypical adenomatous hyperplasia (AAH), a lesion derived from glandular cells in the epithelial tissue of peripheral airways, to adenocarcinoma in situ (AIS), then minimally invasive adenocarcinoma (MIA) before the next step into invasive adenocarcinoma (ADC or IAC) [66]. Early lung carcinoma includes AIS and MIA in the large bronchi. LUSC, which derives from squamous cells primarily in the large bronchi of the central airways, begins with the transformation of normal epithelium to hyperplasia (either mucous or basal). Metaplasia is followed by various degrees of dysplasia, leading finally to severe dysplasia, resulting in carcinoma situ and invasive cancer [67]. Pathways and premalignant lesions for LUSC and LUAD are significantly different. The development of LUSC is characterized by a series of molecular abnormalities that start with histologically normal epithelium and progress through various stages, including metaplastic and dysplastic changes, and ultimately culminate in LUSC. However, AAH is a significant premalignant lesion in the development of LUAD, AAH is implicated in the linear progression of the cells from the terminal respiratory unit to adenocarcinoma in situ (AIS) [68].

Several studies have focused on genetic and epigenetic changes associated with the histological progression of LUAD (Figure 1B). For example, Sivakumar et al. found *BRAF* mutation in 23% of AAH cases, predominantly LUAD cases co-occurring with *EGFR* [69]. However, *KRAS* mutations were limited to *BRAF*-mutant cases and revealed in 18% of AAH, all occurring among ever-smoker patients. Furthermore, the same investigation found (*UBE2C*, *REL*) and (*MAX*) were associated with *KRAS*-mutant AAHs [69]. Many studies have tried to identify whether genetic changes differ in specific states of LUAD. Nevertheless, AIS and early invasive adenocarcinoma share many common gene mutations together, but early invasive ADC exhibits significantly more mutations compared to AIS [70]. For instance, Jia et al. found significant differences in the genetic changes between AIS and MIA concerning *EGFR* mutation frequency, *p53* and *Ki67*, and *cyclinD1* expression level, notably higher in MIA than in AIS [71]. In addition, *EGFR* mutation is a significant subclone in AIS, MIA, and ADC but a minor one in AAH [72]. Other studies also obtained consistent results, representing the mutation frequency of *EGFR* increased from AIS to MIA and remained constant between MIA and IAC during stepwise progression [73]. Nevertheless, the AIS stage does not reveal *ALK* fusions or *ROS1* mutation. In contrast, Haga et al. reported that *EGFR* mutations are similar in early and advanced adenocarcinoma stages, but *EGFR* mutation occurs in the early stage, causing an accumulation of many somatic mutations [74]. Many driver genes in early stages have also been identified in the previous literature reviews, such as *MET* (Y1021N, exon 14 splice site, exon 14 deletions), *BRAF* (A489_Q493del), *KRAS* (G12A, G12D), *ALK* (EML4-ALK) fusions, *RET* (KIF5B-RET), *ERBB2* (V659E), and *MAP2K1* (E102_I103del), with similarity to those in advanced stages [75,76,77,78,79]. This implies that the most essential somatic driver genes are present at an early stage. In addition, the *KRAS*, *NF1*, and *TP53* mutation frequencies were significantly elevated from AIS to MIA and invasive ADC, supporting their role in cancer progression [70]. Gene mutations of *MAP3K14*, *MAP2K1*, and *EGFR* L858R were identified in AAH, MIA, and IAC, respectively [80]. In advanced stages of LUAD, additional oncogenic amplification and tumor suppressor deletion occurred, including a copy number increase in the regions of *MYC* and *TERT* while a loss in *TP53* and *CDKN2A* [74].

Progressive epigenetic changes also occur in AAH and elevate it even further to LUAD, specifically promoter hypermethylation of hallmark cancer genes such as *p16*, *TIMP3*, *DAPK*, *MGMT*, *RARβ*, *RASSF1A*, and *hTERT* [81]. Nevertheless, the hypomethylation of *GORASP2*, *ZYG11A*, and *SFN* genes is much less frequent in invasive adenocarcinoma, which correlates with poorer patient outcomes [82]. Another study found that hypermethylation and hypomethylation of CpG sites increased in the later stages of lesions (AIS, MIA, and IAC) compared to AAH and normal lung tissue [83]. In addition, a high frequency of methylation heterogeneity and loci with distinct methylation were observed in later stages. Generally, the later-stage lesions have more epigenetic changes and fewer genetic alterations, specifically in DNA methylation, compared to early stages [83]. Alterations in chromatin remodeling and RNA splicing, involving the SWI/SNF chromatin-remodeling genes *SMARCA4* and *SMARCA2*, are observed in advanced LUAD stages [74]. Nevertheless, the loss-of-function mutation in the *RBM10* gene, which is involved in RNA splicing, occurs in the early stages of LUAD.

## 5. The Nuclear Genetic Alterations in Lung Cancer Subtypes and Their Racial Distribution

Lung cancer is classified into histological and molecular subtypes, which are of utmost importance for the decision-making process regarding treatment options and disease prognosis or diagnosis [1]. Indeed, both histopathological classification and molecular subtyping of lung cancer play essential roles in predicting the likelihood of bone metastasis [84]. Moreover, molecular subtyping is essential for targeted therapy. For instance, determining the differential expression of genes (DEGs) and microRNAs in lung cancer types may facilitate the treatment or diagnosis at earlier stages [85]. Wang et al. examined pathway enrichments and DEGs across SCLC and NSCLC (both SCC and ADC), highlighting the unique molecular responses to treatment in each cancer type [86]. The 2021 WHO classification for thoracic tumors also updated the molecular abnormalities associated with lung cancer, highlighting key driver mutations and their clinical relevance. *EGFR* mutations are common in Asians and non-smokers, particularly those with LUAD. Conversely, patients with a history of smoking often have *KRAS* mutations, more commonly seen in LUAD. *ALK* rearrangements are more prevalent in younger, non-smoking individuals with LUAD, while *ROS1* fusions have emerged as a novel driver mutation in NSCLC. Additionally, emerging molecular targets such as *RET* mutations, though present in a small percentage of NSCLC cases, reflect the increasing appreciation for the heterogeneity of genetic alterations driving lung carcinogenesis [87].

Background racial disparities in the diagnosis, management, and outcome of lung cancer have persisted in healthcare for decades [88]. Thus, the death rate after lung cancer resection is affected by different races, and genetic changes would differ between ethnicities as well [89]. While disparities in human studies contribute to differences in tumor behavior and response to treatments, so do biological properties of lung cancer cells, such as heterogeneity and the interactions between the tumors and their microenvironment [90]. A further study conducted on NSCLC patients reported that the incidence of *EGFR* mutations is substantially greater among East Asian ancestry, while *KRAS* and *STK11* alterations are more prevalent in EAs and AAs [91]. In contrast, James et al. showed that mutations in *EGFR* are more frequent in AA, while *TP53* mutation is more common in Latin ancestries [92]. Lung disparity-related studies among AA men revealed that they have higher rates of lung cancer incidence and death compared with individuals from all other racial/ethnic groups, likely due to a combination of genetic, socioeconomic, and healthcare access factors [93,94].

### 5.1. The Nuclear Genetic Alterations in LUAD Subtype and Its Racial Distribution

The most common form of NSCLC is LUAD, and it encompasses several precursor lesions: AAH, AIS, MIA, and IAC (Figure 1A) [95]. Genome-wide mRNA expression profiles were generated for the four LUAD molecular subtypes, subtype 4 of which has the best prognosis and the higher rate of *EGFR* mutation. In contrast, subtypes 1 and 2 are related to immune-related processes and *TP53* mutations, suggesting late-stage cancer; subtype 3 showed enrichment in cell cycle dysregulation, and subtype 4 had an extracellular matrix organization signature [96]. Additionally, 379 DEGs and 67 differential methylated sites have identified two clinically relevant subtypes within LUAD [97]. A recent study provided additional information about the mutations in distinct subtypes of lung cancer. Tlemsani et al. demonstrated that the clinical profiles of LUAD patients with *NF1* deletions differed from those with *NF1* mutations, consequently proposing molecular and clinical subclassifications for LUAD [98]. In addition, *KEAP1*/*NRF2*-mutant LUADs can be further grouped into three molecular subtypes, and the two variants of *KEAP1*/*NEFR2*-mutation handle distinct genetic, clinicopathologic, differentiation as well as immunological properties [99]. LUAD is primarily defined by multiple nuclear genetic defects such as *EGFR*, *KRAS* mutant, and *ALK* gene rearrangement, exhibiting significant variation among racial and ethnic populations (Table 1) [100,101]. A recent study showed that EA is positively associated with *KRAS* G12C mutation and negatively associated with *EGFR* mutation [102]. Similarly, Shi et al. found that mutation in *KRAS* G12C is the most common among EAs [101]. On the contrary, East Asians, Hispanic/Latino patients, and individuals with American Indigenous (AMR) ancestry showed the opposite [97,98]. Furthermore, though driver *CTNNB1* mutations are rarely found in non-Hispanic White patients, they are confined to a subgroup of never-smoker non-Hispanic Asian individuals and, more specifically, enriched among those with East Asian ancestry [97]. Shi et al. found a higher rate of *EGFR* exon 21 L858R mutation, *RET* rearrangements, and *ERBB2* amplifications in Asians than in other ethnic groups [101]. In addition, *STK11* mutations are more common in EAs and AAs than in Asians. Ji et al. showed that *ATM* L2307F mutation is more frequent among Ashkenazi Jewish populations [103]. Meanwhile, *EGFR* and *KRAS* mutation frequencies were significantly correlated with genetic ancestry in LUAD patients from Latin America (LA), indicating a strong correlation between germline genetics and the development of mutations in LUAD [104]. Zhang et al. compared the frequency of *EGFR* and *KRAS* mutations among LUAD East Asian and EA patients, suggesting that common oncogenic driver mutations occur more frequently in East Asians with LUAD [105]. The most common driver is *EGFR*, followed by *KRAS* in East Asian patients, whereas the reverse is true for EA patients [104]. Unlike Japanese, *KRAS*, *TP53*, *BRAF*, *PIK3CA*, *KEAP1*, *NF1*, *STK11*, *RBM10*, and *MET* mutations were seen more frequently in EA patients [53]. On the other hand, Tunisian-LUAD patients were found to have a lower percentage of *EGFR* and *KRAS* mutations and *ALK* translocation compared with European or Asian series [106]. Research on tumor suppressor genes (TSGs), such as *TP53*, *STK11*, and *MGA* mutations, revealed a high prevalence of *MGA* in East Asians, whereas *TP53* and *STK11* mutations are more common in EAs [104]. Besides detecting the racial disparities of driver gene mutations, a recent study also found race-specific miRNA isoforms in EA and AA-LUAD patients, highlighting the racial disparities in miRNA expression profiles [107]. Indeed, novel targeted therapies and individualized care must be informed by race/ethnicity-specific lung cancer nuclear genomic alterations.

### 5.2. The Nuclear Genetic Alterations in LUSC Subtype and Its Racial Distribution

The second most prevalent NSCLC histological subtype is LUSC, which is commonly attributed to smoking and chronic inflammation [108,109]. LUSC provides complex mutational nuclear genetics with dramatic frequency heterogeneity among racial groups (Table 1). In India, 5.8% of patients with LUSC were found to harbor *EGFR* mutations, a higher percentage in comparison to EA patients [110]. EA has significantly more *TP53* and *PIK3CA*, *KEAP1*, and *NFE2L2* mutations than Japanese patients [53]. AA patients featured increased homologous recombination deficiency (HRD), higher rates of *PTEN* deletion, and *KRAS* amplification, suggesting that the higher prevalence of homologous recombination deficiency (HRD) is crucial for genomic instability in Blacks [46].

### 5.3. The Nuclear Genetic Alterations in Adenosquamous Carcinoma Subtype and Its Racial Distribution

The term adenosquamous carcinoma (ASC) refers to the presence of mutations associated with both adenocarcinoma and squamous cell carcinoma [111,112]. The nuclear genetic alterations in ASC exhibit significant variation among racial groups (Table 1). For instance, Vassela et al. found *EGFR* and *PI3K* pathway mutations to be the most frequent in ASC patients, while *KRAS* mutation occurs less frequently among EAs [112]. Wang et al. showed that *TP53* and *EGFR* mutations are characteristic events in ASC patients, and *CDKN2A*, *TERT*, and *LRP1B* mutations also recurrently mutate [113]. Also, 64 gene fusions have been described, with *ALK* fusion being the most frequent, followed by *CD74-ROS1* and *ROS1-SYN3* fusions. A pilot study also found that the key driver mutations in ASC are *EGFR* and *MET* [114].

### 5.4. The Nuclear Genetic Alterations in SCLC and Its Racial Distribution

SCLC is the most aggressive type of carcinoma and has a poor prognosis [115]. Rudin et al. identified four molecular subtypes of SCLC, including ASCL1, YAP1, NEU-ROD1, and POU2F3, based on the level of expression of the following transcription factors: achaete-scute homolog1, yes-associated protein 1, neurogenic differentiation factor 1, and POU class2 homeobox 3, respectively. The four types are SCLC-A (ASCL1-dominant), SCLC-Y (YAP1-dominant), SCLC-P (POU2F3-dominant), and SCLC-N (NEUROD1-dominant) [116]. Ding et al. replicated the same categorization of four SCLC molecular subtypes [117]. Furthermore, the four SCLC molecular subtypes were identified based on distinct molecular and clinical features, particularly the finding that the endothelial-to-mesenchymal transition (EndMT) in the SCLC-I subtype is associated with platinum resistance and poor prognosis. Conversely, SCLC-A and SCLC-N subtypes were platinum-sensitive [118]. Curiously, Miyakawa et al. highlighted the critical role of super-enhancer-mediated miRNA expression regulation in determining SCLC molecular subtypes [119]. *TP53*/*RB1* alteration and amplification of *MYC* family genes (*MYC*, *MYCL*, and *MYCN*) are prevalent in SCLCS [120]. Indeed, Pongor et al. have demonstrated that extrachromosomal DNA (ecDNA) contributes to *MYC* gene amplifications in SCLC [121]. In addition, other common mutations are identified in SCLC, including *KMT2D*, *PTEN* and *NOTCH* receptors, and *CREBBP* [120]. Indeed, different subtypes of SCLC are identified based on specific transcription factors transcription, such as ASCL1 (SCLC-A), NEUROD1 (SCLC-N), POUF23 (SCLC-P), YAP1 (SCLC-Y), and a recent acknowledged inflammatory/immune-related gene expression subtype SCLC-I subtype [120]. A comprehensive analysis of 3600 SCLC patients identified rare genetic subsets, including *STK11*-mutant tumors (1.7%) and *TP53/RB1* wild-type tumors (5.5%) [122]. However, *TP53*/*RB1* wild-type tumors did not demonstrate a tobacco mutational signature and exhibited alternate mechanisms of p53/Rb pathway inactivation (*CDKN2A*, *CCND1*, *MDM2*) and high human papillomavirus (*HPV*) positivity. Next-generation sequencing has identified gene mutations in SCLC, such as *LRP1B*, *MAP3K13*, *MSH6*, and *SPEN* [123]. Recent studies found significant variation in nuclear genetic alterations among different racial populations (Table 1). For example, the *TP53* and *RB1* gene mutations are identified as the most common mutations in Chinese patients with SCLC, and other mutations are detected as *LRP1B*, *FAM135B*, *SPTA1*, *KMT2D*, *FAT1*, and *NOTCH3*, emphasizing the ethnicity-dependent mutational profile in Chinese SCLC patients [124]. Similarly, another study in China revealed that *TP53*, *RB1*, and *KMT2D* are the most common mutations in Chinese patients with SCLC, and other novel genes (*LRRK2*, *BRCA1*, *PTCH1*, *ARID2*, and *APC*) are observed in 90% of these patients [125]. *ERBB2* and *CREBBP* gene mutations were identified as the most prevalent genetic alterations in SCLC, followed by *TP53* mutations [126]. However, the additional functionality of identification of this histological phenotype of lung cancer is not just at a genomic level but perhaps more effectively at transcriptomic levels, emphasizing that cell proliferation pathways (example: E2F, G2M, and MYC), upregulated in SCLC and large-cell neuroendocrine cancer as compared to LUAD [127]. In addition, Hu et al. reported that co-mutation of *TP53* and *RB1* and mutations in Wnt/Notch signaling pathways are more prone to be detected in EA patients with SCLC than in Chinese people [128].

## 6. The Mitochondrial Alterations in Lung Cancer and Racial Disparity

**Figure 2:**
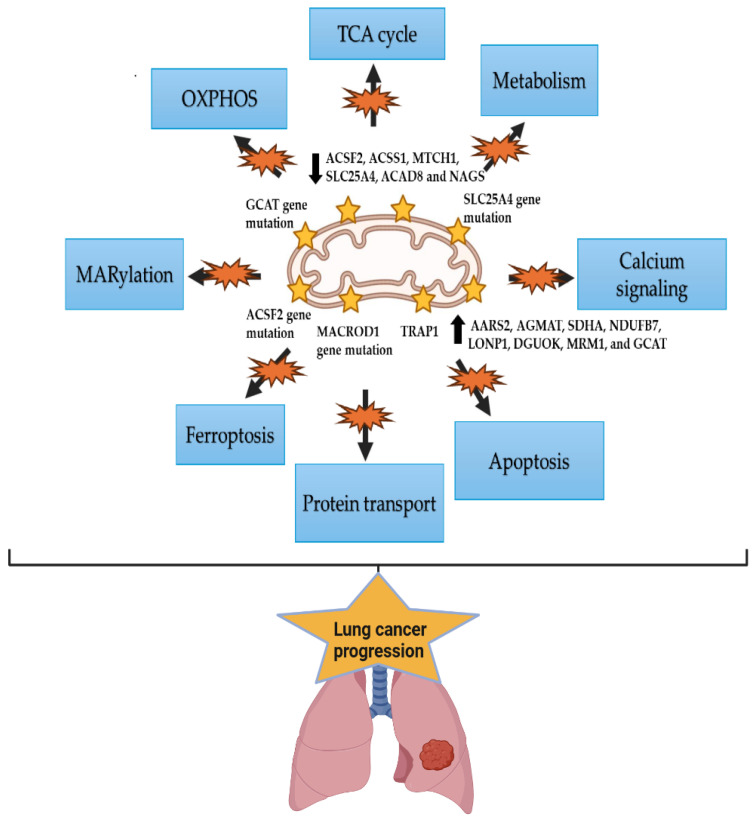
Mitochondrial alterations and the associated functional reprogramming in lung cancer. Altered expressions of genes that predominantly function in mitochondria, including *TRAP1*, *MACROD1*, *SLC25A4*, *ACSF2*, *GCAT*, *AARS2*, *AGMAT*, *SDHA*, *NDUFB7*, *LONP1*, *DGUOK*, *MRM1*, and *GCAT*, *ACSF2*, *ACSS1*, *MTCH1*, *SLC25A4*, *ACAD8*, and *NAGS*, leads to the rewiring of various mitochondrial functional pathways and hence cellular processes. Consequently, these mitochondrial alterations stimulate lung cancer growth and progression. The figure was created with https://www.biorender.com/ (accessed on 13 November 2024).

Cancer cells undergo mitochondrial stress because of engagement in uncontrolled cell proliferation and produce ROS, causing damage to mitochondrial DNA (mtDNA) and mitochondrial proteins, including components of the oxidative phosphorylation (OXPHOS) family, consequently leading to mitochondrial dysfunction [129]. Furthermore, cancer cells activate the mitochondrial stress response to mitigate dysfunction of the mitochondria and aggregation of proteins, which ultimately stimulate the growth and progression of tumors (Figure 2). Mitochondrial genetic changes are also widely present in the occurrence of lung cancer with different ethnic characteristics [130]. Genomic and clinical studies have identified a strong correlation between mitochondrial genomic alterations and lung cancer development and prognosis, as evidenced by copy number variations in mitochondria-targeted genes such as *SLC25A4*, *ACSF2*, and *MACROD1* in NSCLC [131]. Moreover, Yuan et al. observed that more than 5% of lung tumors have somatic transfer of mtDNA into the nucleus, contributing to somatic mutations [132]. Indeed, Hertweck et al. identified 40 mitochondrial-targeted genes and their genetic alterations in NSCLC, including LUAD and LUSC [130]. Altered mitochondrial genes play key roles in ferroptosis, protein transport, apoptosis, calcium signaling, metabolism, the TCA cycle, OXPHOS, and MARylation. In addition, specific alterations in genes (*MACROD1*, *SLC25A4*, *ACSF2*, and *GCAT*) are associated with poor survival in patients. Overexpression was observed for *AARS2*, *AGMAT*, *SDHA*, *NDUFB7*, *LONP1*, *DGUOK*, *MRM1*, and *GCAT*, and reduced expression of *ACSF2*, *ACSS1*, *MTCH1*, *SLC25A4*, *ACAD8*, and *NAGS* in both LUAD and LUSC. A pilot study showed that the mitochondrial heat shock protein TRAP1 induces cisplatin resistance in lung cancer cells and promotes ROS-dependent mitochondrial dysfunction, causing apoptosis inhibition [133]. Similar results were obtained by Kuchitsu et al. [134]. However, another study found that TRAP1 level is low in SCLC patients compared to NSCLC patients, suggesting using TRAP1 in combination with MSA and mad2 for better SCLC diagnosis [135]. Interestingly, mitochondrial alterations linked to lung cancer metastasis include a reduction in mitochondrial membrane potential and overall mitochondrial functionality. These changes were observed in metastatic lung tumor cells compared to the non-metastatic counterparts [136]. Most importantly, the clinical outcomes of early-stage ADC could be predicted by studying mitochondrial DNA (mtDNA), providing a reliable tool for improving patient care [137]. A higher relapse-free survival (RFS) was observed in patients with somatic mutations at the D-loop region, whereas a lower RFS was recorded in patients with respiratory complex (RC) IV and RCV gene mutations [138]. Potentially, the most mutated non-coding region in both somatic and germline mutations is the D-loop region. For protein-coding genes, *CYTB* and *ND* genes had the highest mutation frequency for germline and somatic mutations, respectively. Kazdal et al. found that a significant majority (90.6%) of somatic mtDNA mutations resulted in a non-synonymous amino acid change mapping to a protein-coding gene [139]. Similarly, Jin et al. found that the majority of mtDNA polymorphisms were indeed in protein-coding regions [140]. Moreover, 56 somatic mutations were detected in 60% of the lung cancer patients they analyzed, consisting of 48-point mutations, four single-nucleotide insertions, and four deletions. Interestingly, mtDNA mutations were found to be enriched in never-smoker NSCLC patients compared to current smokers, with a significant association observed between mtDNA and *EGFR* gene mutations [141]. Notably, the majority of the coding mtDNA mutations targeted RCI. In terms of racial disparities, the prevalence of mtDNA mutations was higher in the never-smoker Asian population compared to the current-smoker EA population. In another study, the airway mucosal biopsies obtained from follow-up NSCLC patients were histopathologically negative but exhibited multiple clonal mtDNA mutations consistent with those detected in the corresponding tumors [142].

## 7. The Epigenetic Alteration Patterns in Lung Cancer Patients with Various Ethnic and Racial Backgrounds

Significant racial disparities have been identified in lung cancer patients concerning epigenetic alterations. Different epigenetic modifications have been examined in NSCLC, such as histone modifications, non-coding RNA expression, and DNA methylation [143]. Indeed, DNA methylation has been investigated in lung cancer to understand its role in cancer initiation, progression, and outcome [144]. For instance, a study reported that the DNA hypermethylation of three cancer-related genes (*MTHFR*, *RASSF1A*, and *CDKN2A*) is influenced by tobacco smoking level or gender in lung cancer patients [145]. Understanding these epigenetic alterations in different racial backgrounds is crucial for developing tailored treatments and improving clinical outcomes among lung cancer patients [146].

## 8. The Microbiome Signature in Lung Cancer Subtypes and Racial Differences

Many laboratories have examined microbiome signatures in lung cancer (Table 2). Collectively, recent studies suggest that the human lung microbiome may contribute to lung cancer initiation and progression through various mechanisms, such as bacterial toxin-induced host genomic instability, inducing host inflammatory pathways, altering the local immune environment, the release of cancer-promoting microbial metabolites, and regulation of cancer-related signaling pathways in lung cells [147,148,149]. Nevertheless, a particular oral microbiota, *Leptotrichia* sp._oral_taxon_225, is associated with a reduced risk of developing lung cancer, suggesting its potential protective role [150]. Lung cancer patients have reduced microbial diversity compared to cancer-free individuals. Moreover, they show significant alterations in the abundance of specific bacteria [151,152]. For instance, the abundance of *Actinobacteria phylum*, *Corynebacteriaceae*, *Halomonadaceae families*, *Corynebacterium*, *Lachnoanaerobaculum*, and *Halomonas genera* were remarkably decreased in lung cancer individuals compared to healthy controls [153]. In fact, the racial/ethnic disparities in different subtypes of lung cancer may provide valuable insight for understanding pathogenesis and biomarkers from various microbiome studies [154].

Some specific microbial signatures were found to be associated with lung cancer patients, such as *Enterococcus*, *Lactobacillus*, *Escherichia*, *Phylum TM7*, *Capnocytophaga*, *Blautia*, *Streptococcus*, *Neisseria*, and *Prevotella* [155,156]. Another study suggested microbiota as potential biomarkers for predicting recurrence or metastasis (RM) in certain patients as significant differences have been observed for the presence of *Acidovorax*, *Clostridioides*, *Succinimonas*, and *Shewanella* between RM and non-RM groups [157]. Several studies also reported a significant difference in the abundance of 13 types of bacteria between LUAD and LUSC patients [158,159,160,161]. For instance, Jang et al. determined that *Actinomyces graevenitzii* is more common in LUSC. By contrast, *Haemophilus parainfluenza*, *Neisseria subflava*, *Porphyromonas endodontics*, *Fusobacterium nucleatum*, and *Pseudomonas* are more prevalent in LUAD [161]. In addition, specific bacterial genera, such as *Acidovorax* and *Veillonella*, were found to be potential biomarkers for detecting and discriminating LUSC from LUAD [162]. In LUAD, *Streptococcus* and *Neisseria* are the most prevalent, followed by *Veillonella*. Similarly, *Streptococcus* is the most common in LUSC, followed by *Veillonella* [156]. According to a recent study, *Bacillus* and *Castellaniella* were enriched in the microbiota of patients with LUAD, whereas *Brucella* was predominant during LUSC [163]. In addition, the microbiota diversity is higher in LUSC than in LUAD, particularly among heavy smokers and men, where *Proteobacteria* further discriminated between LUAD and LUSC [164]. Genus *Thermus* and Gram-positive bacteria are significantly more abundant in LUAD than in LUSC [165,166]. Additionally, five intratumoral microbiota, including *Pseudoalteromonas*, *Luteibacter*, *Caldicellulosiruptor Loktanella*, and *Serratia*, have been found to be altered from early to advanced stages of the LUADs [167].

**Table 2:** The microbiome signature and its significance in lung cancer. This table presents the specific abundance of various microbial species and their potential role in lung cancer.

Microbiome	Significance	Citations
*Actinobacteria phylum*, *Corynebacteriaceae*, *Halomonadaceae families*, *Corynebacterium*, *Lachnoanaerobaculum*, and *Halomonas genera*	Decreased in lung cancer patients compared to control people	[153]
*Enterococcus*, *Lactobacillus*, *Escherichia*, *Phylum TM7*, *Capnocytophaga*, *Blautia*, *Streptococcus*, *Neisseria*, and *Prevotella*	Bacterial markers in lung cancer	[155,156]
*Acidovorax*, *Clostridioides*, *Succinimonas*, and *Shewanella*	Prediction of recurrence or metastasis (RM) lung cancer tissue	[157]
*Actinomyces graevenitzii*	Abundant in LUSC	[161]
*Haemophilus parainfluenza*, *Neisseria subflava*, *Porphyromonas endodontics*, *Fusobacterium nucleatum*, and *Pseudomonas*	Abundant in LUAD	[161]
*Acidovorax* and *Veillonella*	Differentiating between LUSC and LUAD	[162]
*Streptococcus* and *Neisseria*	Most prevalent in LUAD	[156]
*Streptococcus*	Most prevalent in LUSC	[156]
*Bacillus* and *Castellaniella*	Enriched in LUAD	[163]
*Brucella*	Enriched in LUSC	[163]
*Proteobacteria*	Discriminated in LUAD and LUSC	[164]
*Thermus* and Gram-positive bacteria	The prevalence is higher in LUAD than in LUSC	[165,166]
*Leptotrichia* sp._oral_taxon_225	Reducing lung cancer risk in African Americans (AA)	[150]

## 9. The Immune Alteration Signature in Lung Cancer

### 9.1. Immune Alteration Signatures in NSCLC and SCLC

**Figure 3:**
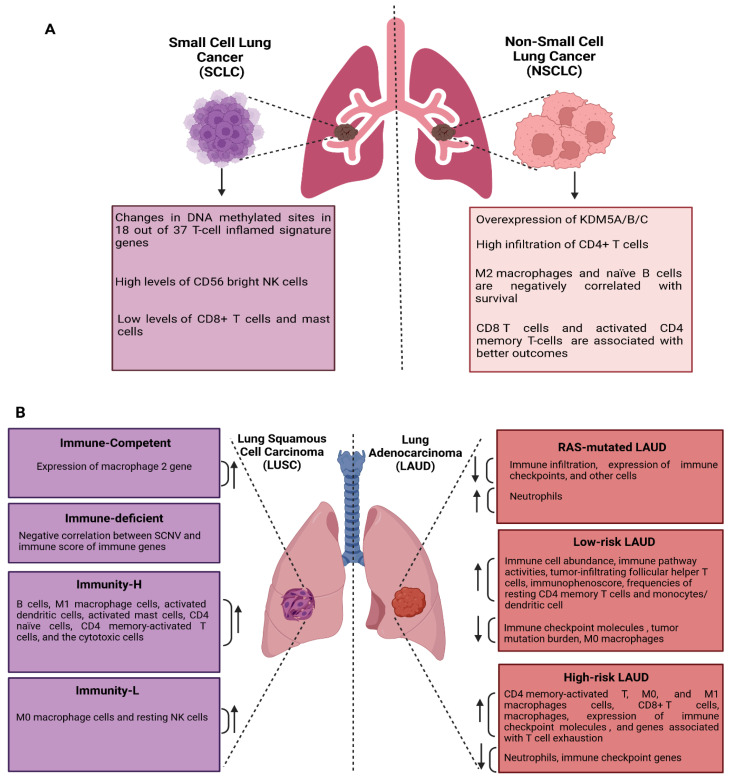
The immune alteration signatures in lung cancer. (**A**) The immune alteration signature in non-small-cell lung cancer (NSCLC) exhibits overexpression of KDM5A/B/C, higher infiltration of CD4+ T cells, M2 macrophages, and naïve B cells have been shown to negatively correlate with survival, whereas CD8 T cells and activated CD4 memory T cells are associated with improved outcomes. The immune alteration signature in small-cell lung cancer (SCLC) exhibits 18 out of 37 T-cell inflamed signature genes associated with changes in DNA methylated sites, high levels of CD56 bright NK cells, and diminished levels of CD8+ T cells and mast cells. (**B**) The immune alteration signatures differ among several lung adenocarcinoma (LUAD) subtypes, including *Ras*-mutated LUAD, low-risk LUAD, and high-risk LUAD. Additionally, squamous cell carcinoma (LUSC) shows different immune alteration signatures among several subtypes, including immune-competent, immune-deficient, high immunity (immunity-H), and low immunity (immunity-L). SCNV: somatic copy-number variation. The figure was created with https://www.biorender.com/ (accessed on 3rd November 2024).

The immune alteration signature in lung cancer is complex and multifaceted, requiring detailed investigation. In fact, immune-based identification differentiates lung cancer and controls patients by analyzing monocytic myeloid-derived suppressor cells (MDSCs), polymorphonuclear MDSCs, intermediate monocytes, and CD8+PD-1+ T cells [168]. Many immune alteration signatures have been reported in NSCLC (Figure 3A). For instance, overexpression of KDM5A/B/C is linked to increased infiltration of CD4+ T cells, especially regulatory T cells (Tregs) and Th17 cells [169]. Han et al. reported that high levels of M2 macrophages and naïve B cells correlate with poor survival, whereas CD8 T cells and activated CD4 memory T cells correlate with better outcomes in NSCLC [170]. Indeed, a study in NSCLC non-responders to immune checkpoint inhibitors (ICIs) identified an immune signature characterized by increased transcriptional activity in the NF-kB and STAT3 pathways, along with a higher level of CD4+ regulatory T cells, resident memory T cells, and TH17 cells [171]. In contrast, the ICI responders exhibit a higher abundance of activated CD8+ T cell subsets. In addition, many immune alteration signatures have been reported in SCLC (Figure 3A). Only 18 out of 37 T-cell inflamed signature genes were associated with changes in DNA methylated sites. This includes hypermethylation at *CCL2*, *CD4*, *IFNG*, and *TNF*, which may contribute to non-inflamed tumor microenvironment (TME) phenotypes and reduced efficacy of immune checkpoint inhibitors (ICIs) [172]. Furthermore, ten gene signatures (*NR3C1*, *NR1D2*, *TANK*, *ARAF*, *HDGF*, *INHBE*, *LRSAM1*, *PLXNA1*, *PML*, and *SP1*) were identified as predictors of overall survival of SCLC patients [173]. This signature is associated with increased immune cell infiltration, characterized by elevated levels of CD56 bright NK cells and reduced levels of CD8+ T cells and mast cells.

### 9.2. Immune Profiles of LUAD and LUSC

The LUAD and LUSC subtypes exhibit different immune alteration signatures (Figure 3B). The analysis of whole exosomes and transcriptomes of LUSC identified two types: immune competent or immune deficient subtypes. The immune-competent subtype exhibited high expression of M2 macrophage signature genes. In contrast, the immune-deficient subtype exhibited a negative correlation between somatic copy-number variation (SCNV) and immune score of immune genes [174]. Li et al. divided LUSC patients into high immunity (immunity-H) and low immunity (immunity-L) groups based on eight immune-related gene signatures, including *C4BPB*, *FCAMR*, *GRAPL*, *MAP1LC3C*, *MGC2889*, *TRIM55*, *UGT1A1*, and *VIPR2*. These signatures could predict overall survival and clinical characteristics [175]. Indeed, the immunity-H subgroup of LUSC showed a high prevalence of B cells, M1 macrophage cells, activated dendritic cells, activated mast cells, CD4 naïve cells, CD4 memory-activated T cells, and cytotoxic cells. On the other hand, M0 macrophage cells and resting NK cells are more prevalent in the immunity-L subgroup. The tumor immune microenvironment is vital in predicting the clinical outcomes of LUSC patients. Resting memory CD4 T cells, naive B cells, follicular helper T cells, and M2 macrophages were associated with the overall survival of these patients [176]. Similarly, one study has found T follicular helper cell initiative to be a prognostic signature for the survival of lung LUSC patients [177]. Moreover, the full-scale research on tumor microenvironment in LUSC identified an immune signature consisting of five genes, including filamin-C, Rho GTPase 1, interleukin 4-induced gene-1, transglutaminase 2, and prostaglandin I2 synthase, which are useful for predicting immunotherapy prediction [178]. The LUAD subtype also shows a distinct immune alteration signature (Figure 3B). For instance, a piolet study discovered 16 genes *EREG*, *HPGDS*, *TSPAN32*, *ACSM5*, *SFTPD*, *SCN7A*, *CCR2*, *S100P*, *KLK12*, *MS4A1*, *INHA*, *HOXB9*, *CYP4B1*, *SPOCK1*, *STAP1*, and *ACAP1* can be utilized to forecast the prognosis of LUAD based on immune cell infiltration in tumor microenvironment (TME) [179]. Another study identified five immune-related genes as potential prognostic markers for LUAD, including *PD1*, *PDL1*, *CTLA4*, *HHLA2*, and *VTCN1* [180]. Similarly, eight immune-related genes associated with prognosis in LUAD are *S100A16*, *FGF2*, *IGKV4-1*, *CX3CR1*, *INHA*, *ANGPTL4*, *TNFRSF11A*, and *VIPR1* [181].

In addition, Song et al. discovered an immune-related gene signature for LUAD, including *MAL*, *MS4A1*, *OAS1*, and *WFDC2* genes, which can distinguish patients at high or low risks [182]. *RAS*-mutated LUAD also exhibited decreased immune infiltration and reduced expression of immune checkpoints. In addition, a significant decrease in other cells was observed, such as B cells, CD8+ T cells, dendritic cells, natural killer cells, and macrophages [183]. *RAS*-mutated LUAD is associated with an increase in neutrophils, which impairs the activity of cytotoxic lymphocytes and antigen presentation. Chen et al. classified LUAD patients into high- and low-risk groups based on an immune-related lncRNA signature. Many low-risk patients exhibit higher levels of immune cell abundance, increased immune pathway activity, and lower immune checkpoint molecules than the high-risk patients [184]. Moreover, Li et al. classified LUAD patients into low and high-risk groups based on seven immune-related gene signatures, including *CD1B*, *CHRNA6*, *CLEC12B*, *CLEC17A*, *CLNK*, *INHA*, and *SLC14A2*, which could predict overall survival and clinical characteristics in these patients [175]. Moreover, CD4 memory-activated T cells and M0 and M1 macrophages associated with poor prognosis tended to distribute into LUAD patients with high-risk gene signatures. On the other hand, low-risk patients demonstrated higher frequencies of resting CD4 memory T cells and monocytes/dendritic cells, correlating with a good prognosis [185]. A similar study showed that LUAD patients with low-risk scores demonstrated better overall survival, elevated tumor-infiltrating follicular helper T cells, low levels of M0 macrophages, lower tumor mutation burden, and higher immunophenoscore [186]. Analyzing genes involved in cell death pathways also significantly stratified LUAD patients as high and low-risk groups [187]. Moreover, the high-risk group is characterized by increased infiltration of CD8+ T cells and macrophages, higher expression of immune checkpoint molecules (*CD-274*, *PD-L1*, and *CTLA-4*), and increased level of T cell exhaustion (*HAVCR2*, *TIGIT*, *LAG3*, *PDCD1*, *CXCL13*, and *LYN*). Additionally, neutrophil infiltration was lower. Two molecular subtypes of LUAD, iC1 and iC2, are identified by multi-omics analysis, with *PTTG1*, *SLC2A1*, and *FAM83A* as signatures for these subtypes [188]. The iC2 has a high Tumor Immune Dysfunction and Exclusion (TIDE) score, representing an immune-suppressive state characterized by elevated levels of CD8+, activated CD4+ cells, and PD-L1 expression, resulting in poor outcomes. The LUAD patients without *EGFR*, *ALK*, *ROS1*, and *BRAF* mutations are enriched with an immune-related prognostic model (IPM) based on three immune-related genes (*PDE4B*, *RIPK2*, and *IFITM1*) [189]. The IPM also identified the high-risk group by low expression in immune checkpoint genes (*CTLA-4*, *PDCD1*, *HAVCR2*, and *TIGIT*). Moreover, Immune checkpoint genes were also used to identify immune alteration signatures, composed of *FCER2*, *CD200R1*, *RHOV*, *TNNT2*, *WT1*, *AHSG*, and *KRTAP5-8*, to classify patients with LUAD into low and high-risk groups based on survival rate and immunotherapy responsiveness [190]. Additionally, a B cell signature combined with gene expression predicts the survival of LUAD patients. Overall survival is positively correlated with an increasing percentage of B cells [191]. A tumor microenvironment immune alteration signature including eight prognostic genes (*ATAD5*, *CYP4F3*, *CYP4F12*, *ESPNL*, *FXYD2*, *GPX2*, *NLGN4Y*, and *SERPINC1*) was found to be effectively predicting the overall survival of LUAD patients [192]. This eight-gene signature group has significantly poorer overall survival, characterized by higher levels of naive B cells, plasma cells, T cell follicular helper, M1 macrophage, and lower levels of T cells CD4 memory resting, monocytes, and resting dendritic cells.

## 10. Future Perspectives

Going forward, it is crucial to overcome the limitations of current lung cancer diagnostic tests, such as LDCT and chest X-rays, which suffer from low sensitivity and specificity. To this end, the development of non-invasive or minimally invasive biomarkers is of great necessity to improve early diagnosis and prognosis. For instance, liquid biopsy assay using blood, bodily fluids, or derived extracellular vesicles (EVs) bear promise for lung cancer detection [193]. In addition, circulating tumor cells (CTCs) and circulating tumor DNA (ctDNA) offer noninvasive methods for early diagnosis, monitoring treatment response, and prognostic evaluation [194]. Moreover, the research gap in microbiome analysis across different racial and ethnic groups must also be addressed in concert with genetic and epigenetic analysis. Along this path, determining the role of mitochondrial genetic and biological alterations in lung cancer and racial disparity is warranted, considering the essential role of mitochondria in human tumorigenesis. Additionally, little is known about how epigenetic changes (e.g., DNA methylation and histone modifications) differ between various racial and ethnic groups, which may aid in developing more tailored treatments. Although many studies have investigated nuclear genetic alterations in LUAD and LUSC among different ethnic groups, very little is known regarding the complete genotype of these genes in less-studied histologic subtypes such as ASC and SCLC subtypes. Closing these research gaps is crucial for better diagnostics, prognosis, and therapeutically improved outcomes. Application of emerging technologies such as spatial genomics, single-cell sequencing, characterization of circulating tumor cells and tumor cell DNA, CRISPR, and non-CRISPR-based genome editing tools in concert with artificial intelligence (AI) systems is likely to improve our understanding of lung cancer initiation and progression in various racial populations. The emerging application of AI in lung cancer screening and detection reduces radiologists’ workload, suggesting improved patient outcomes and supporting the integration of AI into routine clinical practice [195]. In parallel, the generation of race-specific organoid or animal model systems to understand the complex cellular and molecular interplay within the tumor microenvironment is warranted.

Globally, the overall reductions in lung cancer incidence are undermined by substantial disparities in marginalized individual populations, highlighting the urgent need to extend access to quality care [196]. Additionally, the benefits of recent treatment advances have not been equitably evident in different groups [44]. Solving those inequities requires public health policies to invest more in education, screening programs, and community outreach.

## 11. Conclusions

This review provides a comprehensive overview of the complexities that underlie lung cancer in terms of biology, genetics, and environmental influences, with particular reference to racial and ethnic disparities. It also illustrates the progress in understanding molecular and clinical characteristics of lung cancer and their histological subtypes, including LUAD and LUSC. On the other hand, it also highlights the considerable disparity in research on rarer subtypes of lung cancer, such as ASC and SCLC, particularly when it comes to divergent racial populations. It has been demonstrated that genetic mutations, epigenetic modifications, and microbiome dysbiosis contribute to race-specific differences in lung cancer initiation and progression and response to standard anti-cancer therapy. The review also highlights how screening technologies and other present diagnostic procedures are limited in terms of early detection, monitoring, and guiding therapeutics of lung cancer with precision. Minimizing these research gaps and enhancing diagnostic and prognostic accuracy is key to moving towards more effective, personalized treatments that benefit all patients, regardless of their race or ethnicity. These results reinforce the need for additional research and creativity in deconstructing lung cancer, with a specific eye on bridging outstanding survival gaps within cancer care.

## Figures and Tables

**Table 1:** The nuclear genetic alterations in different lung cancer types and their racial distribution. This table exhibits nuclear genetic changes in small-cell lung carcinoma and non-small-cell lung carcinoma with various histologic subtypes including squamous cell carcinoma, adenosquamous carcinoma, and lung adenocarcinoma.

Lung Cancer Types	Race Information	Nuclear Genetic Alterations
Small-cell lung cancer (SCLC)	Chinese	*TP53* and *RB1* gene mutations are the most prevalent *LRP1B*, *FAM135B*, *SPTA1*, *KMT2D*, *FAT1*, and *NOTCH3*
	EA	Co-mutation of *TP53* and *RB1* Wnt and Notch signaling pathways mutations
Squamous cell carcinoma (NSCLC)	EA	*TP53*, *PIK3CA*, *KEAP1*, and *NFE2L2* mutations
	Indian	*EGFR* mutations
	AA	Increased homologous recombination deficiency (HRD) Higher rates of *PTEN* deletion and *KRAS* amplification
Adenosquamous carcinoma (NSCLC)	EA	Less prevalent *KRAS* mutation
Adenocarcinoma (NSCLC)	EA	Positively associated with *KRAS* G12C mutation Negatively associated with *EGFR* mutation *STK11* mutations The common driver is *KRAS*, and the second is *EGFR* *TP53*, *BRAF*, *PIK3CA*, *KEAP1*, *NF1*, *STK11*, *RBM10*, and *MET* mutations
	East Asian, Hispanic/Latino, and American Indigenous (AMR)	Negatively associated with *KRAS* G12C mutation Positively associated with *EGFR* mutation
	Never-smoker non-Hispanic Asian, specifically East Asian ancestry	*CTNNB1* driver mutations
	Asian	*EGFR* exon 21 L858R mutation *RET* rearrangements *ERBB2* amplifications
	AA	*STK11* mutations
	LA	*EGFR* and *KRAS* mutations
	EA and AA	Specific miRNA isoforms
	Ashkenazi Jewish	*ATM* L2307F mutation
	Tunisian	Reduced frequency of *EGFR* and *KRAS* mutations and *ALK* rearrangement

## Data Availability

Not applicable.
